# LATE ONSET NODULAR PRURIGO – THE SOLE AND INITIAL MANIFESTATION OF OCCULT HODGKIN'S DISEASE

**DOI:** 10.4103/0019-5154.53182

**Published:** 2009

**Authors:** Vandana Mehta, Aarti Sarda, C Balachandran, Raghavendra Rao, Puja Monga

**Affiliations:** *From the Departments of Skin and STD, Kasturba Medical College, Manipal, Karnataka, India. E-mail: vandanamht@yahoo.com*

Sir,

Hodgkin's lymphoma, also known as Hodgkin's disease, was first described by Thomas Hodgkin in 1832. It is unique among the nodal lymphomas for its onset in young adults, characteristic presence of CD30+/CD15+ Reed-Sternberg cells, and the pattern of spread to contiguous lymph nodes.[1,2] Cutaneous involvement in Hodgkins disease usually occurs only in the setting of advanced nodal or visceral disease. We report one such case of nodular prurigo that remained undiagnosed for 2 years till we discovered a coexisting Hodgkin's Lymphoma in the same patient.

**Figure 1:**
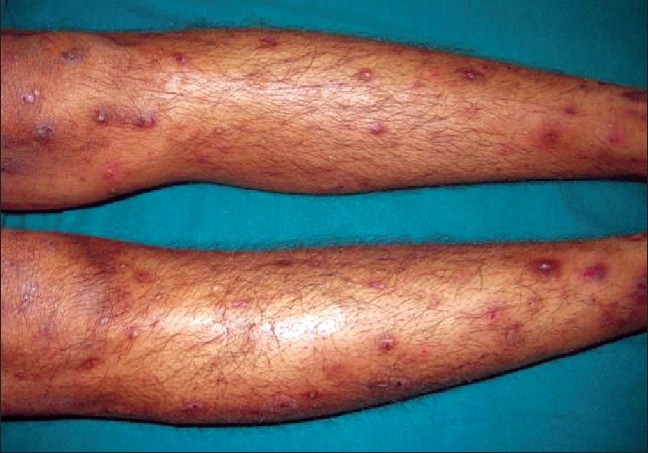
Clinical picture showing the hyperpigmented papulonodules on the lower limbs

**Figure 2:**
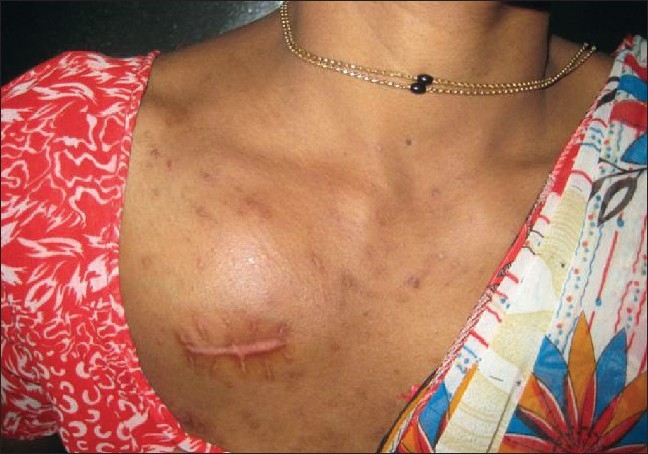
Infraclavicular asymptomatic swelling

A 35-year-old female presented to us with itchy papulonodular lesions reminiscent of prurigo nodularis over the trunk and extremities of two years duration, for which she was treated with several medications without any benefit. On further questioning, she gave a history of weight loss for the past three months. On examination, she was found to be pale and her right axillary lymph node was enlarged. Cutaneous examination revealed multiple hyperpigmented papules, plaques, and nodules over the trunk, upper, and lower extremities [Figure 1]. A firm fixed nodular swelling of 8 × 8cm in Rt. infraclavicular region was an incidental finding [Figure 2]. The CT scan of thorax revealed an irregular heterogenous mass extending from inferior margin of clavicle to lower margin of third rib with bony destruction of anterior aspects of first and second rib. The inview of this suspicious swelling in the infraclavicular region and enlarged axillary lymph node an FNAC was performed which showed abnormal large cells with abundant amphophilic cytoplasm, vesicular indented nucleus, and coarse chromatin suggesting the possibility of nodular sclerosis variant of Hodgkin's Lymphoma, which was subsequently confirmed on histopathology of an incisional biopsy specimen.

Unlike other lymphomas whose incidence increases with age, Hodgkin's lymphoma has a bimodal incidence curve. It occurs most frequently in two separate age groups, the first being young adulthood (15–35 years) and the second being those over 55 years old. Overall, it is more common in males, except for the nodular sclerosing variant, which is more common in females. Skin lesions associated with Hodgkin's disease may occur in 17–35% of patients and are usually paraneoplastic findings. Cutaneous manifestations of Hodgkin's disease can be divided into specific and nonspecific. Specific lesions such as papules, infiltrated plaques, nodules, and ulcerative lesions are rare and develop in 0.5–3.4% of patients respectively and have specific histologic features of Hodgkin's disease.[3] Their mode of spread could be by hematogenous dissemination, retrograde flow through lymphatics, contiguous extension of the underlying nodal focus to the skin, and by primary cutaneous involvement.[4] Nonspecific lesions such as pruritus, prurigo, urticaria, erythroderma, erythema nodosum, acquired ichthyosis, eczema, alopecia, and pigmentation are more common and develop in 10–50% of patients with Hodgkin's disease.[7]

Although pruritus is a common symptom of Hodgkin's disease, prurigo nodularis has rarely been reported as a Hodgkin's symptom. Till date, only two cases have been described in English language literature.[5,6] Our patient was a young lady with nodular prurigo in whom a Hodgkin's lymphoma went unnoticed for many years. It also illustrates the need for an extensive malignancy workup in any patient presenting with adult onset prurigo, which is refractory to treatment.
